# An outbreak investigation of scrub typhus in Nepal: confirmation of local transmission

**DOI:** 10.1186/s12879-021-05866-6

**Published:** 2021-02-18

**Authors:** Meghnath Dhimal, Shyam Prakash Dumre, Guna Nidhi Sharma, Pratik Khanal, Kamal Ranabhat, Lalan Prasad Shah, Bibek Kumar Lal, Runa Jha, Bishnu Prasad Upadhyaya, Bhim Acharya, Sanjaya Kumar Shrestha, Silas A. Davidson, Piyada Charoensinphon, Khem B. Karki

**Affiliations:** 1 Nepal Health Research Council (NHRC), Ramshah Path, Kathmandu, Nepal; 2 Central Department of Microbiology, Tribhuvan University, Kathmandu, Nepal; 3 Institute of Tropical Medicine, Nagasaki University, Nagasaki, Japan; 4 Epidemiology and Disease Control Division, Department of Health Services, Ministry of Health and Population, Government of Nepal, Kathmandu, Nepal; 5 Ministry of Health and Population, Government of Nepal, Kathmandu, Nepal; 6 Institute of Medicine, Tribhuvan University, Kathmandu, Nepal; 7 Department of Health Services, Ministry of Health and Population, Government of Nepal, Kathmandu, Nepal; 8 National Public Health Laboratory, Ministry of Health and Population, Government of Nepal, Kathmandu, Nepal; 9 Walter Reed/AFRIMS Research Unit Nepal (WARUN), Kathmandu, Nepal; 10 Armed Forces Research Institute of Medical Sciences (AFRIMS), Bangkok, Thailand

**Keywords:** Scrub typhus, Rickettsial infection, *Orientia tsutsugamushi*, Local transmission, Outbreak, Epidemiology of scrub typhus, Nepal

## Abstract

**Background:**

Scrub typhus is a largely ignored tropical disease and a leading cause of undifferentiated febrile illness in the areas of tsutsugamushi triangle caused by *Orientia tsutsugamushi*. It is frequently diagnosed in South Asian countries, although clear epidemiological information is not available from Nepal. After the 2015 earthquake in Nepal, a sudden upsurge in scrub typhus cases was reported. The objective of this study was to investigate epidemiology of scrub typhus and its causative agents in humans, animals, and chigger mites to understand the ongoing transmission ecology.

**Methods:**

Scrub typhus cases with confirmed diagnosis throughout the country were included in the analysis. Studies were concentrated in the Chitwan district, the site of a major outbreak in 2016. Additional nation-wide data from 2015 to 2017 available from the government database included to analyse the disease distribution by geographical mapping.

**Results:**

From 2015 to 2017, 1239 scrub typhus cases were confirmed with the largest outbreak occurring in 2016 with 831 (67.1%) cases. The case fatality rate was 5.7% in 2015 which declined to 1.1% in 2017. A nationwide outbreak of scrub typhus was declared as the cases were detected in 52 out of the 75 districts of Nepal. Seasonal trend was observed with a peak during August and September. In addition to the human cases, the presence of *O*. *tsutsugamushi* was also confirmed in animals (rodents) and chigger mites (Leptotrombidium imphalum) from the outbreak areas of southern Nepal.

**Conclusion:**

The detection of *O. tsutsugamushi* in humans, animals, and chigger mites from outbreak locations and wide-spread reports of scrub typhus throughout the country consecutively for 3 years confirms the ongoing transmission of *O. tsutsugamushi* with a firmly established ecology in Nepal. The country’s health system needs to be strengthened for systematic surveillance, early outbreak detection, and immediate actions including treatment and preventive measures.

**Supplementary Information:**

The online version contains supplementary material available at 10.1186/s12879-021-05866-6.

## Background

Scrub typhus is a vector-borne acute febrile illness caused by *Orientia tsutsugamushi* and transmitted to humans and rodents by infected chigger mites (larval stage of Trombiculidae mites) [1, 2]. Historically, scrub typhus had been endemic in Asia, Australia and islands in the Indian and Pacific Oceans, known as the “tsutsugamushi triangle” [1]. However, there have been recent reports of scrub typhus from Africa, the Middle East, and South America suggesting the disease is no longer restricted to this triangle [2] but no autochthonous cases have been reported from north America and Europe. Scrub typhus is frequently reported from many Asian countries and is endemic in Nepal’s neighboring countries including India (Sub-Himalayan belt) and Bhutan, where it is considered an emerging infectious disease [3–6]. In Bhutan, one in six undifferentiated febrile patients had rickettsial infections, with scrub typhus being the most common [6]. However, the disease situation in Nepal remained unexplored until 2015, likely due to the high burden of other febrile illnesses with indistinguishable clinical signs and limited availability of diagnostics.

There have been a few previous attempts to investigate the incidence of scrub typhus in Nepal. As early as 1981, a study showed the high possibility of scrub typhus in Nepal when it demonstrated elevated antibody titers in 10% of healthy adults [7]. Unfortunately, additional surveillance studies in the country were not conducted for next 23 years until 2004. A serological investigation of scrub typhus at Patan Hospital, Kathmandu found a small number of febrile patients (28/876) positive for scrub typhus antibodies [8]. The investigation was performed using a multi-test assay without further confirmation for scrub typhus by immuno-fluorescent assay (IFA), gold standard for scrub typhus diagnosis. It was inconclusive whether scrub typhus or another rickettsial illness, such as murine typhus, was present. Another report in 2007 also indicated the presence of scrub typhus in Nepal [9]. However, no outbreak investigations of scrub typhus (with fatality information) were reported in Nepal before 2015 and no systematic investigations by the government had been conducted. As a consequence, scrub typhus cases had not been reported to the Epidemiology and Disease Control Division (EDCD) of the Ministry of Health and Population before 2014 [10].

In April 2015, Nepal experienced a mega-earthquake claiming thousands of lives, massive destruction, and huge economic losses followed by an upsurge in febrile illnesses [11, 12]. Three months after this devastating earthquake in Nepal (August 2015), a tertiary care teaching hospital in Nepal alerted EDCD that children with fever and severe respiratory features were not responding to the usual course of antibiotic treatment, leading to high mortality rates (8%) [10, 11]. The clinicians had used cefexime or ceftraiaxone and imipenem in intensive care. After the initially suspected aetiologies (hantavirus and other viral diseases) were ruled out, the samples were screened with M- Enzyme Linked Immunosorbent Assay (ELISA) for scrub typhus and found positive. This was the first and most significant fatal scrub typhus outbreak in the country [11]. Since then, scrub typhus has been increasingly reported in Nepal but no clear epidemiological picture is available.

In this study, a systematic investigation was carried out in Nepal to investigate the ongoing transmission ecology of Scrub typhus which included patient, vector, and animal studies employing both serological and molecular tools.

## Methods

### Study design and settings

This was a descriptive cross-sectional study conducted in Nepal and included scrub typhus cases of any age and sex reported in 2016 from 52 districts out of 75 districts the country. Nepal has three distinct ecological regions from north to south, namely: mountain, hill and terai (plain area). Muntain region is comprised of high mountains in the north including the top of the world while hilly regions consists of mid-hills between mountains and terai (low-land). Terai region is the southern low-land ecological trails spanning from east to west.

**Fig. 1:**
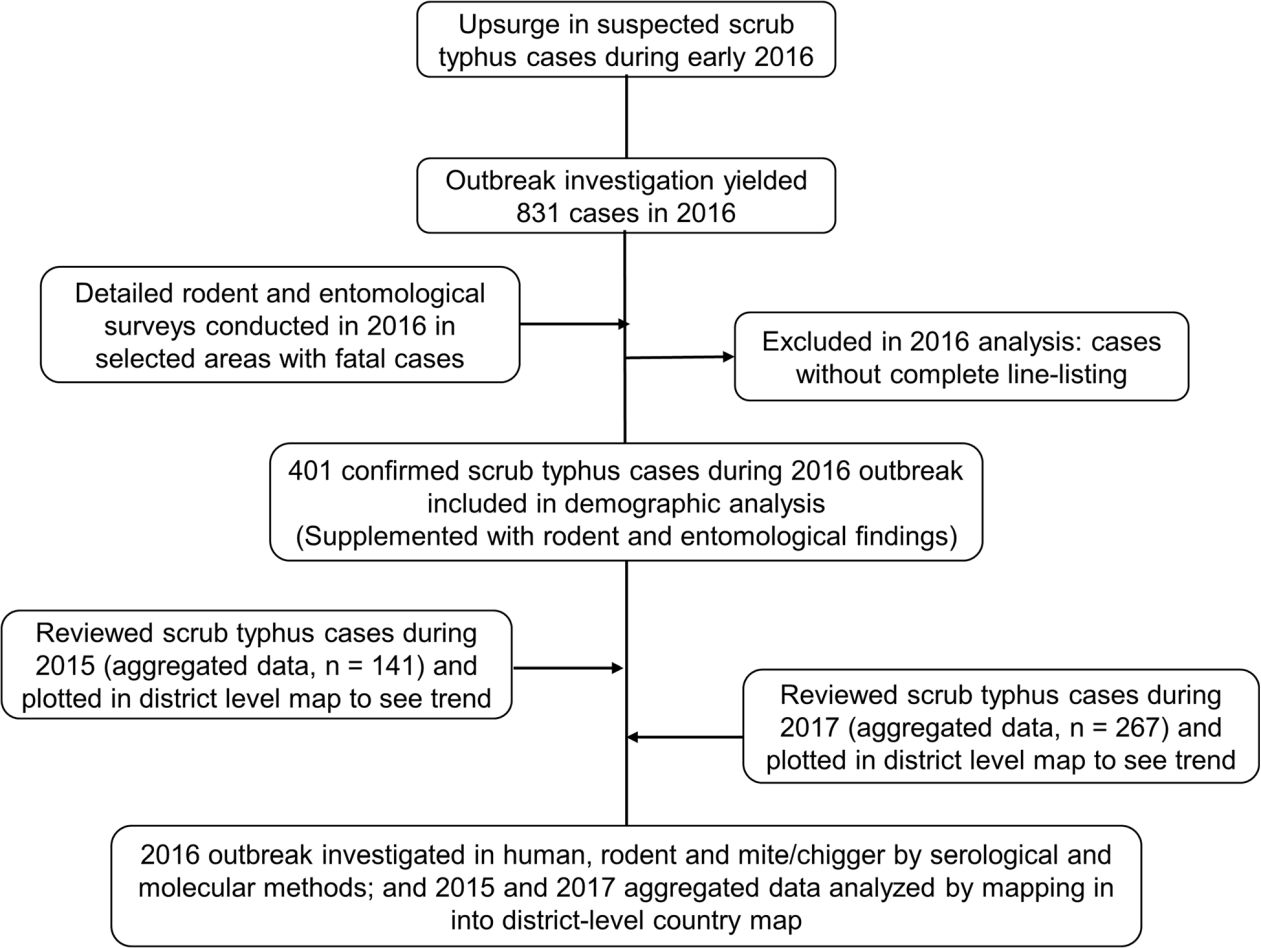
Overall flow-diagram of the study

Serologically confirmed cases of scrub typhus from various government authorized laboratories, including National Public Health Laboratory (NPHL), were reported to EDCD and compiled in the database of EDCD. As part of the outbreak investigation, chigger and animal studies were also carried out in the major outbreak areas in Chitwan district as detailed below. Additionally, the aggregated data from 2015 and 2017 were used for mapping. An overall flow diagram of the study is detailed in Fig. 1.

### Case definitions

The patients were classified based on the EDCD Guideline on Prevention and Control of scrub typhus adopted from the World Health Organization (WHO) [10]. Briefly, 1) a case with acute undifferentiated febrile illness (AUFI) of ≥5 days with or without eschar (fever of < 5 days for a case with eschar) was considered a “*suspected/clinical case”*; 2) a suspected/clinical case with an IgM titer > 1:32 and/or a four-fold increase of titers between acute and convalescent sera was considered to be a “*probable case”*; and 3) a “*confirmed case* of scrub typhus” was declared when the *O. tsutsugamushi* DNA was detected in eschar or whole blood samples by polymerase chain reaction (PCR), or four-fold rise in antibody titers on acute and convalescent sera by IFA, which is the gold standard assay, or Indirect Immuno-Peroxidase (IIP) assay.

### Human sample collection and laboratory investigation for *O. tsutsugamushi* infection

The Scrub Typhus Detect™ IgM Rapid Test kits were used for the detection of IgM antibodies to members of *O. tsutsugamushi* species in human blood of all cases visiting periphery level health care centers. For outbreak investigation purpose, blood samples were collected from the suspected scrub typhus cases presented to the NPHL, Kathmandu, and from Chitwan Medical College (CMC) Hospital and Bharatpur Hospital, Chitwan. Samples collected from sentinel sites including CMC and Bharapur Hospital were transferred to NPHL, maintaining the cold chain. Samples were tested for scrub typhus using an *O. tsutsugamushi* specific IgM-ELISA (Scrub Typhus Detect™ Kit, InBios International, WA, USA) and interpreted as per the manufacturer’s manual. The remaining aliquots of the sample were cryo-preserved at − 80 °C. Representative scrub typhus IgM positive and negative serum samples (confirmed by IgM-ELISA) were further confirmed by IFA using a panel of *O. tsutsugamushi* specific antigens covering major *O. tsutsugamushi* strains identified in endemic areas at the Armed Forces Research Institute of Medical Sciences (AFRIMS), Bangkok, Thailand and Walter Reed/ AFRIMS Research Unit Nepal (WARUN) as described previously [13, 14]. Briefly, pooled antigens from whole cell-cultured prototype strains including Karp, Kato, and Gillian were used to detect *O. tsutsugamushi* specific antibodies in patient serum samples. An initial screen was performed using dilution of 1:50. Any positive sample was then 2-fold serially diluted to a final concentration of 1:12,800 and tested for antibody titer. FITC-conjugated rabbit anti-human IgG (Rabbit anti-human IgG-FITC secondary Antibody) was used to determine *O. tsutsugamushi* specific antibodies in humans. Serum titers less than 1:50 were interpreted as negative for IgM and IgG, whereas serum titers 1:50 and less than 1:400 indicated past or recent infection, and serum titer equal to or higher than 1:400 indicated active infection. This cutoff is a reference value currently used by several research institutes including National Institute of Health (http://nih.dmsc.moph.go.th/login/showimgdetil.php?id=514) and Ministry of Science and Technology, Thailand. The epidemiological characteristics of confirmed patients such as age, sex, ethnicity, month of diagnosis, ecological region, administrative region and clinical outcome was recorded. Out of the total 831 cases reported in 2016, complete demographic data requested was only available for 401 cases.

### Animal and entomological investigation of scrub typhus outbreak

#### Site selection

**Fig. 2:**
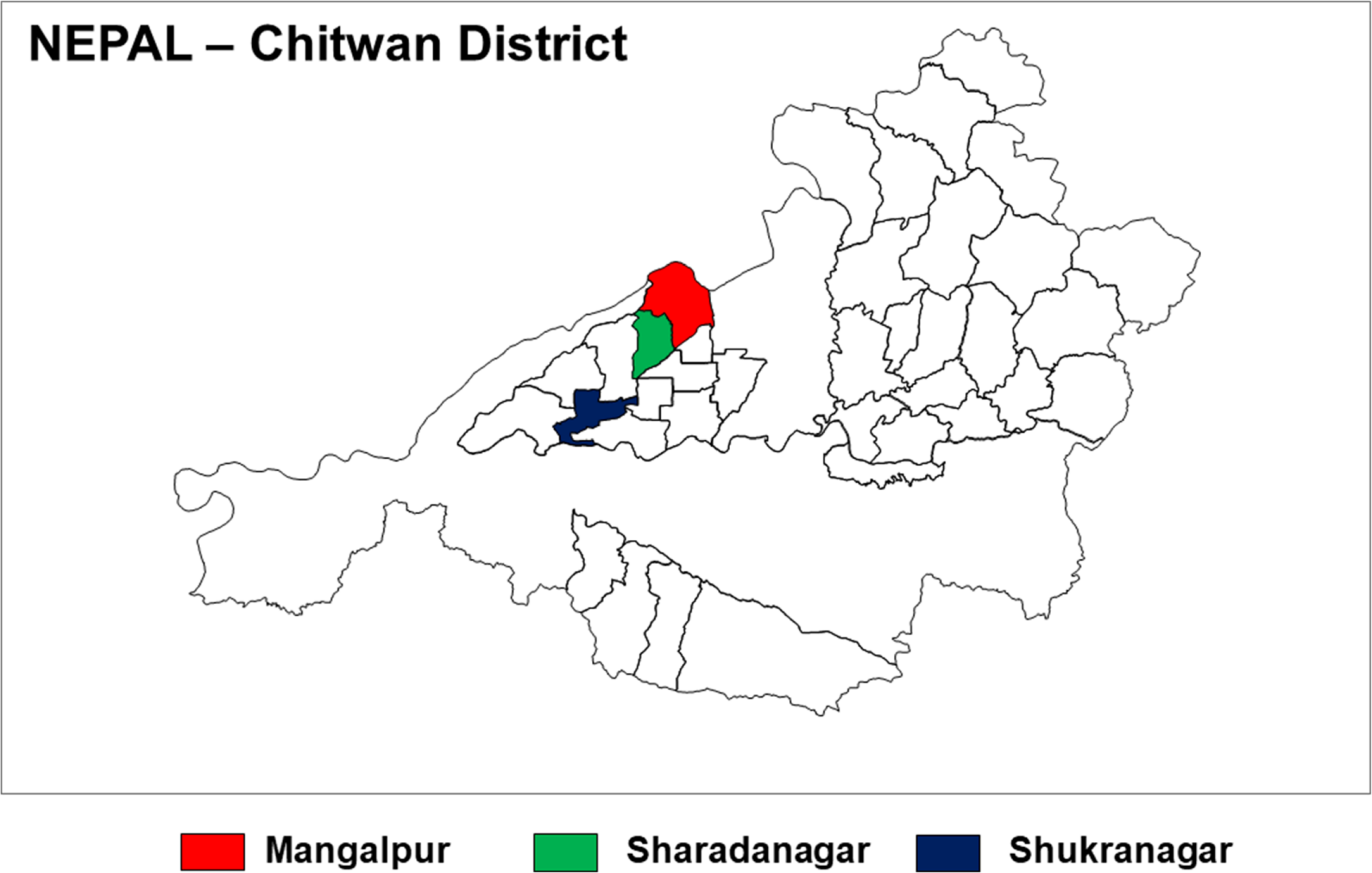
Map of Chitwan district showing the animal and entomological study sites. Investigation was carried out in three VDCs (Mangalpur, Sharadanagar and Shukranagar) (dark filled areas) which are located to the South-West of the district headquarters of Chitwan (the outbreak epicenter district). VDC, village development committee (Source: EDCD data using Arc GIS software)

Chitwan district was selected for animal and entomological surveillance based on the severity and prevalence of the disease in and around this district. Chitwan district belongs to terai region which is the low-land plain region (elevation as low as 70 m above mean sea level) situated at the southern belt of Nepal (south of the outer foothills of Himalayas) and bordering to India. Terai has warmer and humid climate and is endemic for many vector-borne tropical disease. Fatalities due to scrub typhus mainly occurred in the Mangalpur Village Development Committee (VDC), which is a sub-district level administrative area of Chitwan district. Therefore, three VDCs (Mangalpur, Sharadanagar and Shukranagar), which are located to the South-West of Bharatpur (the district headquarters), were selected for the surveillance study (Fig. 2).

#### Animal capture

Rodent and shrew trapping was performed in and around the houses of the index cases and their neighbors. They were trapped using Sherman traps tagged with a unique number for identification as described previously [14]. All the traps were equipped with different baits (ripe banana, tomato, or piece of chicken) and placed at various sites (*n* = 104) inside and outside houses, cattle sheds, near granaries and the nearby fields (e.g. kitchen gardens) following the trap lines. Traps were set in the early evening and collected the following morning. A total of 104 traps were placed and 12 animals (nine rodents and three shews) were captured.

#### Animal blood collection and species identification

The animals, still in the trap, was placed inside a gas chamber and anaesthetized using carbon dioxide inhalation method (2–3 L/h, maintaining 30% initial CO_2_). Following the euthanasia, the animal was taken out from the trap, weighed, and essential characteristics were recorded for species identification. Blood was collected by direct cardiac puncture using 3 ml disposable syringes. Serum samples pipetted into cryo-tubes were kept in a cold box with pre-frozen ice packs at − 80 °C and immediately shipped to the laboratory of WARUN at Bharatpur Hospital and then to WARUN in Kathmandu in cold box where the samples were stored at − 80 °C until analyzed. The samples were analyzed at WARUN for further related investigations. Rodent and shrew species were identified by their physical appearance, morphology, length of head and body, tail as well as ear/pinna, color patterns of dorsal and ventral fur, number of mammary glands.

### Mite collection, identification and processing

Mites and other ectoparasites were collected by combing the anaesthetized animal’s outer surfaces (ventral and dorsal surfaces, arm-pits, groins, and areas near pinnas etc.) onto a clean, white sheet of paper to help identify the ectoparasites. The ear pinna of animals was examined under a dissecting microscope for chiggers and individual or clusters of chiggers were removed together with a thin layer of ear skin using fine forceps. Chiggers were transferred into labelled vials containing 70% ethanol and stored in an ice-cold box for preservation. Representative chiggers from each animal host were randomly sampled for morphological identification. Permanent slides of chigger samples were prepared and mounted at WARUN laboratory in Kathmandu, Nepal following the method described previously [15]*.* The animal sera were tested for the presence of IgG antibody against *O. tsutsugamushi* by IFA. Moreover, the *O. tsutsugamushi* specific quantitative PCR (qPCR) as performed in animal tissue samples and chigger samples as described previously [14].

### Data analysis

Data were entered in Microsoft Excel and analyzed in IBM SPSS version 22.0. Descriptive analysis was done by calculating frequency and percentages for categorical data. Data were analyzed by chi-square tests for assessing significant difference in proportions across groups. To understand the nationwide situation of scrub typhus, cases were plotted onto the district-level country maps.

### Ethics

This study was approved by the Ethical Review Board of Nepal Health Research Council (NHRC), Government of Nepal (Reg. No 305/2016) which approves health research involving human and animal subjects in Nepal. This study was conducted following National Ethical Guidelines for Health Research in Nepal and Standard Operating Procesuure 2011 and Ethical Guidelines for the Use and Care of Animals in Health Research in Nepal 2005. All patient records were anonymized before analysis. All procedures involving animals were carried out as per the Ethical Guidelines for the Care and Use of Animals in Health Research in Nepal- 2005 and followed the regulation of local and national laws regarding the use of animals for research purpose [16].

## Results

### Socio-demographic findings

**Fig. 3:**
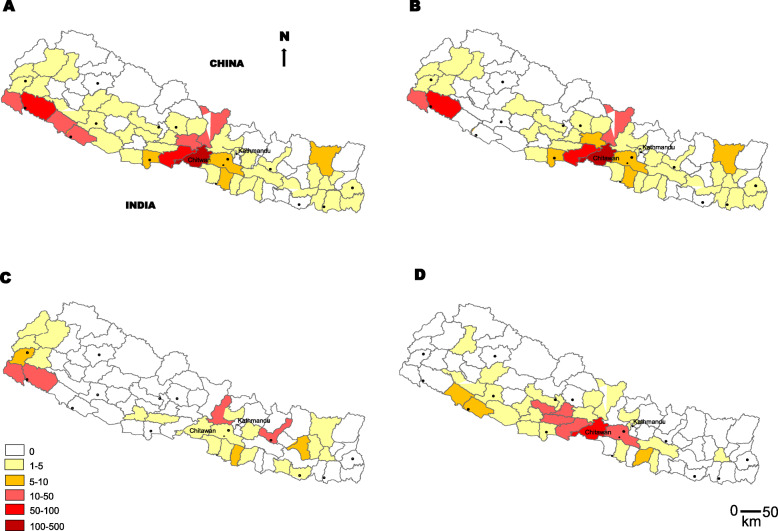
Distribution scrub typhus cases during major outbreaks in Nepal. In 2016, a total of 831 scrub typhus cases (based on peripheral reporting) were reported from 47 of the 75 districts (**a**), of which a total of 401 cases from 42 districts were confirmed at national reference laboratory and included in further analysis (**b**), while 141 scrub typhus cases from 25 districts were reported to EDCD in 2015 based on aggregated data available from EDCD (**c**), and a total of 267 cases were confirmed from 30 districts in 2017 (**d**). Respectively 8, 14 and 3 deaths due to scrub typhus were confirmed in 2015, 2016 and 2017. From 2015 through 2017, 52 out of 75 districts of the country reported cases of and/or deaths due to scrub typhus (Source: EDCD data using Arc GIS software)

In 2016, a total of 831 cases with 14 deaths (case fatality rate (CFR) = 1.7%) were reported from 47 districts throughout the country (Fig. 3). Although 831 cases were reported in the country during April to December 2016 in Nepal, a complete line listing was available for only 401 cases (male = 163; female = 238) from EDCD. The mean age (years) ± standard deviation of the cases was 28.9 ± 18.2 years and median was 25.0 (IQR 14.0–42.0) years. More than half of the cases (57.1%) were below 30 years of age; of these 14.5% were children below 10 years of age (Table 1). The majority of the cases belonged to *Janajati/Aadhibasi* (44.4%) and *Brahmin/Chhetri* (44.1%) ethnic groups with no gender-based differences.

**Table 1:** Epidemiological characteristics of scrub typhus cases in Nepal, 2016*

Variables	Male n (%); ***n*** = 163	Female n (%); ***n*** = 238	Total n (%); ***n*** = 401	***p***-value
**Age in years, median (IQR)**	24.0 (11.0–50.0)	25.0 (17.0–39.0)	25.0 (14.0–42.0)	0.237
**Age group**				
Below 10	37 (22.7)	21 (8.8)	58 (14.5)	< 0.001
10–19	37 (22.7)	51 (21.4)	88 (21.9)	
20–29	19 (11.7)	64 (26.9)	83 (20.7)	
30–39	16 (9.8)	44 (18.5)	60 (15.0)	
40–49	11 (6.7)	21 (8.8)	32 (8.0)	
50–59	28 (17.2)	25 (10.5)	53 (13.2)	
60 and above	15 (9.2)	12 (5.0)	27 (6.7)	
**Children vs adults**				
15 years and below	63	54	117	0.001
Above 15 years	100	184	284	
**Ethnicity**				
Brahmin/Chhetri	72 (44.2)	105 (44.1)	177 (44.1)	0.87
Janajati/Aadhibasi	74 (45.4)	104 (43.7)	178 (44.4)	
Madheshi	5 (3.1)	5 (2.1)	10 (2.5)	
Dalit	9 (5.5)	18 (7.6)	27 (6.7)	
Others	3 (1.8)	6 (2.5)	9 (2.2)	
**Month (seasonality)**				
June	12 (7.4)	5 (2.1)	17 (4.2)	0.01
July	27 (16.6)	29 (12.2)	56 (14.0)	
August	71 (43.6)	93 (39.2)	164 (41.0)	
September	36 (22.1)	69 (29.1)	105 (26.2)	
October	17 (10.4)	41 (17.3)	58 (14.5)	
**Ecological region**				
Mountain	8 (4.9)	2 (0.8)	10 (2.5)	0.03
Hill	28 (17.2)	37 (15.5)	65 (16.2)	
Terai	127 (77.9)	199 (83.6)	326 (81.3)	
**Administrative region**				
Eastern	12 (7.4)	6 (2.5)	18 (4.5)	0.08
Central	75 (46.0)	107 (45.0)	182 (45.4)	
Western	39 (23.9)	56 (23.5)	95 (23.7)	
Mid-Far West	37 (22.7)	69 (29.0)	106 (26.4)	
**Clinical outcome**				
Cured	157 (96.3)	232 (97.5)	389 (97.0)	0.50
Death (CFR)	6 (3.7)	6 (2.5)	12 (3.0)	

*Scrub typhus patients with complete line-listing information (*n* = 401) were included in this analysis

*IQR* Inter-quartile range; *CFR* Case fatality rate

Overall, young adults and children were the majority affected by scrub typhus in Nepal, and this proportion significantly differed by gender (*p* <  0.001) (Table 1). Among male scrub typhus patients, the age skewed equally towards children below 10 years (22.7%) and 10–19 years (22.7%) while among females, more cases were in the age group 20–29 years (26.9%). There was no difference in the median age of scrub typhus patients by gender (*p* = 0.237) (Table 1).

### Geographical and temporal distribution of scrub typhus in Nepal, 2016

The vast majority of scrub typhus cases were from Tarai (81.5%; *p* = 0.03), the low-land ecology of southern Nepal bordering northern India. The regional analysis identified the central region (45.6%) as the most affected area in the country (Table 1, Fig. 3). The most affected district was Chitwan, contributing 34.4% of the total cases (*n* = 138), followed by Kailali (*n* = 63), Nawalparasi (*n* = 55), Kanchanpur (*n* = 26), Gorkha (*n* = 15) and Tanahun (*n* = 10) (Fig. 3, Suppl Table 1). The 2016 outbreak of scrub typhus demonstrated a seasonal trend in Nepal that peaked in the months of August (*n* = 164; 40.9%) and September (*n* = 105; 26.2%) (*p* = 0.01) (Table 1).

### Diagnosis, outcome and trend of scrub typhus cases in Nepal, 2015–2017

In 2016, cases were reported or confirmed at five health facilities (Suppl Table 1) mostly from the outbreak epicenter, Chitwan district (61.3%). Representative serum samples (*n* = 61) tested by IFA further confirmed the presence of *O. tsutsugamushi* infection in Nepal (Table 2). From the 33 samples which tested positive by ELISA, 28 also tested positive by IFA. Similarly, one of the 28 ELISA negative sample was positive by IFA. Regarding the prognosis of the disease, 12 cases (3%) (6 males and 6 females) died while the rest recovered after treatment. After analyzing the cases reported to EDCD during 2015 to 2017 (*n* = 1239), it was determined that the disease was firmly established in Nepal, and a large outbreak occurred in 2016 (*n* = 831) involving 47 districts. An additional 267 cases were reported in 2017 and there were 141 reported cases in 2015 (Table 3). Twenty-five people died as a result of scrub typhus during 2015–2017 with an overall CFR of 2.0, which decreased from 5.7 in 2015 to 1.1 in 2017 (Table 3).

**Table 2:** Laboratory findings of selected human samples underwent IFA confirmation

Sample collection sites	Total samples	Positive by RDT (%)	Positive by IgM ELISA (%)	Positive by IFA (%)
NPHL	50	NT	30 (60.0)	26 (52.0)
Bharatpur hospital	11	3 (27.3)	NT	3 (27.3)

A fraction of samples (*n* = 61) from suspected scrub typhus cases were randomly selected and sent to reference laboratory (Armed Forces Research Institute of Medical Sciences, Bangkok, Thailand) for IFA confirmation. *RDT*, rapid diagnostic test; *ELISA* Enzyme linked immune-sorbent assay; *IFA*, Indirect fluorescence antibody assay; *NPHL* National Public Health Laboratory; *NT* Not tested

**Table 3:** Trend of scrub typhus cases and deaths in Nepal, 2015–2017

Year	No. of districts	No. of cases (%)	No. of deaths (%)	Case fatality rate, %
2015	25	141 (11.4)	8 (32.0)	5.7
2016	47	831 (67.1)	14 (56.0)	1.7
2017	30	267 (21.5)	3 (12.0)	1.1
Cumulative, 2015–2017	52	1239 (100)	25 (100)	2.0

### Animal and entomological investigation identified hosts of *O. tsutsugamushi* in the outbreak areas of Nepal

Of the total 104 traps used, 12 animals (9 the *Rattus rattus* species complex and 3 shrews named *Suncus murinus*) were successfully trapped. (Suppl Table 2). Out of 12 animals, three (25%) had chigger mite infestation. The chigger index was 0.92. Details of animals and chiggers have been presented in Suppl Tables 2 and 3.

### Confirmation of *O. tsutsugamushi* infection in animals and chiggers collected in the outbreak areas of Nepal by IFA/ molecular techniques

Three animal serum samples out of nine were confirmed scrub typhus positive by IFA (IgG titer > 50) (Suppl Table 3). However, all the 24 animal tissue samples were *O. tsutsugamushi* negative by PCR. Similarly, one of the three chigger (*Leptotrombidium imphalum*) samples was also confirmed *O. tsutsugamushi* positive by PCR.

## Discussion

Our study demonstrates that scrub typhus is an emerging public health problem in Nepal with several outbreaks since 2015. Scrub typhus is an under studied neglected tropical disease and a leading cause of undifferentiated treatable fever in Asia [1, 17]. This study uncovered the firmly established nature of scrub typhus outbreaks in Nepal through evidence of the causative agent *O. tsutsugamushi* in human (patients), animals and vector/reservoir hosts (chigger mites) during the recent fatal outbreaks.

Despite the frequent reports of outbreaks from neighbouring countries, particularly India [3–6, 18–21], scrub typhus was undetected in Nepal for decades after its first indication in 1981 [7]. This has resulted in a significant lack of understanding when it comes to epidemiological features, treatment response and severity, and its ecological niche. This type of baseline information is required for a country to formulate appropriate guidelines and pave strategies for scrub typhus control. Apart from a handful of reports discussing the human cases [7–10, 22, 23], no study has explored whether the human-host-vector/reservoir-pathogen cycle is maintained in Nepal. This ecological chain is essential to understand a sudden outbreak of any magnitude [24]. The present study has contributed by providing solid evidence of the ongoing circulation of the scrub typhus pathogen *O. tsutsugamushi* among human, animals, and chigger mites in areas affected by recent large outbreaks.

The scrub typhus outbreaks that occurred in Nepal during 2015 to 2017 may be linked to the devastating earthquake of 2015. The outbreaks could have been triggered as a result of intimate contact between humans and mite infested rats that might have come out of their usual underground habitat with the demolition of many houses [25], and this would provide people increased exposure to *O. tsutsugamushi* infected mites which are both the vectors and the reservoirs. However, there is no evidence to support this hypothesis. Close proximity while living in temporary shelters [12] due to overcrowding and unsanitary conditions could have contributed to increased contact between vectors, pathogens and humans [25]. A weakened health system due to the massive earthquake compromised the availability of diagnostic and treatment facilities, which further affected the control program resulting in the large outbreak in 2016.

A large scrub typhus outbreak during 2015 to 2017 initiated in 2015 with 141 cases and nine deaths, which was an unforeseen eruption of this disease after the years of silence in the country. The apparent disappearance of the disease in a territory for a long period before a sudden re-emergence in an epidemic form is symptomatic of scrub typhus. For example, scrub typhus re-emerged in the Maldives as a fatal epidemic in 2002–2003 after 58 years of its disappearance [26, 27], and it also resembles the scenario in India where the re-emergence was observed during 1990s after World War II [4]. There was no strong evidence of persistent scrub typhus in Nepal after the initial indication in 1981, although we cannot totally exclude this possibility considering the lack of diagnostic facilities, endemicity, and inadequate clinical suspicion/precision in Nepal. In peripheral settings of Nepal, widal test is exclusively used for enteric fever due to the lack of blood cultures facilities. This may lead to misdiagnosis of enteric fever [12], one of the most common AUFI [28], when the actual etiology might be other febrile diseases like scrub typhus, leptospirosis, dengue, etc. Such misdiagnosis [12] and the minimal clinical interest (due to effective treatment) could be other factors for no visible infections [26]. Nevertheless, we cannot overlook the serologically positive cases reported in 2004 and 2007 in the Kathmandu valley [8, 9] while considering such absence of the overt disease in this location.

Among those infected with scrub typhus, the majority were females aged below 40 years, with a significant proportion of children. Younger and reproductive females in rural areas are mostly involved in outdoor or agriculture activities in Nepal,and this could be one reason for increased scrub typhus infection. India, South Korea and China have also reported higher incidence among females [18, 29, 30]. Similarly, we observed a clear seasonality with the majority of cases being detected in August and September, which is quite similar to what was reported from the Indian states [18, 21]. The overall pattern of scrub typhus in Nepal resembles that in neighbouring countries including India, indicating potential cross-border transmission due to massive trade and transport activities (woods, trucks) and lack of physical barrier probably provide ample opportunity for chiggers’ migration across the borders .

In Nepal, scrub typhus cases were reported nationwide (52 of 75 districts) in just 3 years, and the disease may expand very rapidly as seen in other parts of the world [31, 32]. In this study, the majority of cases were reported from lowland terai districts where other febrile illnesses including Japanese encephalitis (JE) [33, 34], leptospirosis [35], and dengue [33, 34, 36, 37] have been frequently reported. Even before the initiation of JE vaccination in Nepal, only one-third of the acute encephalitis syndrome (AES) cases were due to JE, which declined with immunization. However the AES remains persistent, clearly indicating other aetiologies of AES in the country. From the same areas of Nepal, cases of leptospirosis and dengue were identified among AES population [34, 35]. Looking at the severity and high fatality (up to 6%) of scrub typhus during 2015 to 2017, it is logical to consider that a fraction of AES cases could be due to scrub typhus and vice-versa in Nepal. This is further supported by the contemporaneous outbreaks occurring in some states of India (along the Nepal border) where scrub typhus was identified as one of the significant causes of AES [20, 24]. Interestingly, six out of eight fatal cases reported in that Indian state had evidence of *O. tsutsugamushi* infection [20]. In addition to AES cases, rodents and mites were also positive for *O. tsutsugamushi* in those AES-reported areas [24]. This is similar to what we found in Nepal, suggesting the need to include scrub typhus in the differential diagnosis in AES and other acute undifferentiated febrile illness (AUFI) which are common in Nepal [38].

Despite the high fatality rate (6%) observed during the first wave of scrub typhus in Nepal in 2015, CFR successfully declined to approximately 1% in 2017, which is quite encouraging for the disease control program. This rate is within the wide range of reported fatality (median, 6 and 1.4% for untreated and treated cases, respectively) [1]. The government initiatives that may have reduced the CFR [10, 11] include distribution of guidelines, public awareness, and training and orientation of health workers for prompt treatment of scrub typhus with available drugs (doxycycline, azithromycin, and chloramphenicol or their appropriate combinations). Infection with resistant or reduced drug-susceptible *O. tsutsugamushi* strains often yield very high mortality (up to 24%), miscarriage and poor neonatal outcomes [1]; it may be speculated that these strains are yet to emerge in Nepal. Since there was no definitive evidence for this, careful monitoring for drug resistance (including potential resistance genes) should be in place. Poor response with doxycycline in some countries suggested the potential emergence of resistant strains [26]. Early administration of doxycycline/azithromycin reduced the progression to AES in India [3], and so treatment delay should be avoided.

This study has some limitations. The detailed individual level data were not available for cases of 2015 and 2017, and even all cases of 2016 was not available for analysis which suggest the need of sustainable integrated surveillance system. The IFA and PCR confirmation tests were limited to representative samples, and further genetic characterization of the pathogenic strain of *O. tsutsugamushi* that circulated in Nepal was not performed. Although a total of 104 animal traps were set in the suspected areas, a relatively small number of animals were captured as well as a low number of chigger mites (*L. imphalum*). We cannot discount the possibility of other animal species associated with scrub typhus in Nepal. Although we did not cover other rickettsial diseases, cases of spotted fever group, typhus group and Q Fever rickettsia in scrub typhus endemic areas of neighbouring countries like India [19] and Bhutan [6] warrant further investigation of these aetiologies in Nepal. Recent indications of Q fever in some acute undifferentiated febrile cases in Nepal also underscores this [22]*. Apart from humans, very high* rickettsial *seropositivity was reported* among domestic animals in Bhutan suggesting that a One Health approach could be useful in understaning the prevalence of *O. tsutsugamushi* through serological sentinel surveys that may aid in rickettsial diseases control programs [39]. Therefore, the need for domestic animal studies is also key in the context of Nepal.

## Conclusion

The outbreak investigation in humans, animals and chigger mites with positive serological and molecular evidence, and an analysis of additional aggregated data confirm a firmly established scrub typhus infection ecology in Nepal. This is evidence of ongoing transmission of *O. tsutsugamushi* in Nepal. Scrub typhus cases were reported throughout the country for 3 years after the 2015 earthquake and this underlines the risk for outbreaks of epidemic potential. Establishment of a sufficient reference laboratory with IFA and molecular facility is urgently required in the country for confirmation, genetic characterization for detection of numerous strains in circulation and evolutionary analysis. Most importantly, the health system needs to be strengthened for systematic surveillance, early outbreak detection and immediate responses including treatment and preventive measures in the country.

## Supplementary Information

**Additional file 1: **Supplementary information on extended methodological details on animal and entomological investigations. Supplementary information on findings of animal and entomological investigations. **Supplementary Table 1.** Distribution of Scrub typhus cases in in Nepal, 2015–2017. **Supplementary Table 2.** Characteristic features of animals captured during scrub typhus outbreak in Nepal. **Supplementary Table 3.** Serological (IFA) and molecular findings of animals and chigger samples during scrub typhus outbreak in Nepal.

## Data Availability

The datasets used and/or analysed during the current study are available from the corresponding author on reasonable request.

## Abbreviations

AES: Acute encephalitis syndrome
AFRIMS: Armed forces research institute of medical sciences
CFR: Case fatality rate
CMC: Chitwan Medical College
EDCD: Epidemiology and Disease Control Division
JE: Japanese encephalitis
IFA: Immuno-fluorescent assay
IgM-ELISA: Immunoglobin M- enzyme linked immunosorbent assay
IIP: Indirect immuno-peroxidase
NHRC: Nepal Health Research Council
NPHL: National Public Health Laboratory
PCR: Polymerase Chain Reaction
VDC: Village Development Committee
WARUN: Walter Reed Afrims Research Unit Nepal
WHO: World Health Organization
