# Supplementary material for: An outbreak investigation of scrub typhus in Nepal: confirmation of local transmission
Source: BMC Infect Dis. 2021 Feb 18;21:193. doi: 10.1186/s12879-021-05866-6 (PMC7893900; doi:10.1186/s12879-021-05866-6)
Supplement: Supplementary file 1 — Additional file 1: Supplementary information on extended methodological details on animal and entomological investigations. Supplementary information on findings of animal and entomological investigations. Supplementary Table 1. Distribution of Scrub typhus cases in in Nepal, 2015–2017. Supplementary Table 2. Characteristic features of animals captured during scrub typhus outbreak in Nepal. Supplementary Table 3. Serological (IFA) and molecular findings of animals and chigger samples during scrub typhus outbreak in Nepal. [file 12879_2021_5866_MOESM1_ESM.doc]

**Supplementary materials**

**An outbreak investigation of scrub typhus in Nepal, confirmation of local transmission**

Meghnath Dhimal ^1,$,*^, Shyam Prakash Dumre ^2,$^, Guna Nidhi Sharma^3,4^, Pratik Khanal^1,5^, Kamal Ranabhat^5,6^, Lalan Prasad Shah^6^, Bibek Kumar Lal^3^, Runa Jha^7^, Bishnu Prasad Upadhyaya^7^, Bhim Acharya^3^, Sanjaya Kumar Shrestha^8^, Silas A. Davidson^9^, Piyada Charoensinphon^9^, Khem B. Karki^1,5^

^1^Nepal Health Research Council (NHRC), Ramshah Path, Kathmandu, Nepal

^2^Institute of Tropical Medicine, Nagasaki University, Nagasaki, Japan

^3^Epidemiology and Disease Control Division, Department of Health Services, Ministry of Health and Population, Government of Nepal, Kathmandu, Nepal

^4^Ministry of Health and Population, Government of Nepal, Kathmandu, Nepal

^5^Institute of Medicine, Tribhuvan University, Kathmandu, Nepal

^6^Department of Health Services, Ministry of Health and Population, Kathmandu, Nepal

^7^National Public Health Laboratory, Kathmandu, Nepal

^8^Walter Reed/AFRIMS Research Unit Nepal (WARUN), Kathmandu, Nepal

^9^Armed Forces Research Institute of Medical Sciences (AFRIMS), Bangkok, Thailand

$ Equal contribution

* Corresponding author

Meghnath Dhimal

Email: [meghdhimal2@gmail.com](mailto:meghdhimal2@gmail.com)

A. **Supplementary information on extended methodological details on animal and entomological investigations**

## **Animal and entomological investigation of scrub typhus outbreak**

**Animal capture:** Rodent and shrew trapping was performed in and around the houses of the index cases and their neighbors. They were trapped using Sherman traps tagged with a unique number for identification as described previously . All the traps were equipped with different baits (ripe banana, tomato, or piece of chicken) and placed at various sites (n = 104) inside and outside houses, cattle sheds, near granaries and the nearby fields (e.g. kitchen gardens) following the trap lines. Traps were set in the early evening and collected the following morning. A total of 104 traps were placed and 12 animals (Nine rodents and three shews) were captured.

### **Animal blood collection and species identification:** The animals, still in the trap, was placed inside a gas chamber and anaesthetized using carbon dioxide inhalation method (2-3 L/h). Following the euthanasia, the animal was taken out from the trap, weighed, and essential characteristics were recorded for species identification. Blood was collected by direct cardiac puncture using 3 ml disposable syringes. Serum samples pipetted into cryo-tubes were kept in a cold box with pre-frozen ice packs at -80°C and immediately shipped to the laboratory of WARUN at Bharatpur Hospital and then to WARUN in Kathmandu in cold box maintaining temperature at -80°C. The samples were analyzed at WARUN for further related investigations. Rodent and shrew species were identified by their physical appearance, morphology, length of head & body, tail as well as ear/pinna, color patterns of dorsal & ventral fur, number of mammary glands.

### **Mite collection, identification and processing**

Mites and other ectoparasites were collected by combing the anaesthetized animal’s outer surfaces (ventral and dorsal surfaces, arm-pits, groins, and areas near pinnas etc.) onto a clean, white sheet of paper to help identify the ectoparasites. The ear pinna of animals was examined under a dissecting microscope for chiggers and individual or clusters of chiggers were removed together with a thin layer of ear skin using fine forceps. Chiggers were transferred into labelled vials containing 70% ethanol and stored in an ice-cold box for preservation. Representative chiggers from each animal host were randomly sampled for morphological identification. Permanent slides of chigger samples were prepared and mounted at WARUN laboratory in Kathmandu, Nepal following the method described previously.

### **Laboratory investigation of non-human samples (animals and chiggers)**

***O. tsutsugamushi* specific antibody detection in rodent serum by IFA assay:** The animal serum samples were tested for the presence of IgG antibody against *O. tsutsugamushi* by IFA assay with a panel of O. tsutsugamushi antigens described previously for the human samples. The seropositivity cut off value for interpreting infection in animal sera for IgG was 1:50.

***O. tsutsugamushi* specific PCR using animal tissue samples:** Lung and liver tissues from animals were used for pathogen detection. Tissue samples were dissected and homogenized in buffer ATL (Qiagen, Hilden, Germany) with sterile 5 mm stainless steel beads and processed using Tissuelyzer LT (Qiagen). The homogenate was lyzed with proteinase K and subjected to DNA extraction using the QIAmp DNA mini kit (Qiagen). Subsequent quantitative polymerase chain reaction (qPCR) amplification for *O. tsutsugamushi* was performed using specific primer targeting 47-kDa membrane protease protein encoding gene (htrA) as described previously . Positive samples were confirmed by amplification of 56-kDa tissue specific antigen gene and the DNA fragment was resolved using a 1% agarose gel electrophoresis and Ultra violet system.

***O. tsutsugamushi* specific PCR using chigger samples:** Each chigger mite was placed in a tiny drop of phosphate buffered saline (pH 7.4) under a dissecting microscope. The exoskeleton and internal tissue contents were separated by a puncture and squeeze method. The chigger exoskeleton was mounted on a slide for species identification, while the internal contents was homogenized and used for PCR identification of *O. tsutsugamushi* using the 56-kDa-TSA gene as described above for animal tissues.

**B. Supplementary information on findings of animal and entomological investigations**

## **Animal and entomological investigation identified hosts of *O. tsutsugamushi* in the outbreak areas of Nepal**

Of the total 104 traps used, 12 animals were successfully trapped. Nine of the 12 were identified as a member of the *Rattus rattus* species complex, while 3 remaining were shrews named *Suncus murinus.* (Table 5). Out of 12 animals, three (25%) had chigger mite infestation. Three animals each had 4, 3 and 3 mites, respectively. The chigger index was 0.92. Details of animals and chiggers have been presented in Tables 5 and 6.

**Confirmation of *O. tsutsugamushi* infection in animals and chiggers collected in the outbreak areas of Nepal by IFA/ molecular techniques**

Three animal serum samples out of nine (all rodents) were confirmed scrub typhus positive by IFA (IgG titer > 50) (Table 6). However, all the 24 animal tissue samples (12 lungs and 12 livers) were *O. tsutsugamushi* negative by PCR, suggesting that no active infection was observed in these population. Similarly, one of the three chigger samples was also confirmed *O. tsutsugamushi* positive by PCR.

**C. Supplementary Tables**

**Supplementary Table 1. Distribution of Scrub typhus cases in in Nepal, 2015-2017**

| **S.N.** | **District** | **2015** | | **2016** | | **2017** | |
| --- | --- | --- | --- | --- | --- | --- | --- |
|  |  | **Cases** | **Deaths** | **Cases** | **Deaths** | **Cases** | **Deaths** |
| 1 | Chitwan | 4 |  | 138 | **4** | **64** | **1** |
| 2 | Kailali | 36 |  | 63 | **4** | 0 | 0 |
| 3 | Nawalparasi | 0 |  | 55 | 0 | **42** | 0 |
| 4 | Kanchanpur | 14 |  | 26 | 0 | 0 | 0 |
| 5 | Gorkha | 0 |  | 15 | 0 | 2 | 0 |
| 6 | Tanahun | 0 |  | 10 | **1** | 5 | 0 |
| 7 | Makwanpur | 3 |  | 9 | 0 | **49** | 0 |
| 8 | Sankhuwasava | 5 |  | 8 | **2** | 0 | 0 |
| 9 | Bara | 3 |  | 6 | 0 | 4 | 0 |
| 10 | Rupandehi | 0 |  | 6 | 0 | 5 | 0 |
| 11 | Dadeldhura | 7 |  | 5 | 0 | 0 | 0 |
| 12 | Dhading | 17 |  | 5 | **1** | 0 | 0 |
| 13 | Sarlahi | 2 |  | 5 | 0 | 6 | 0 |
| 14 | Kavrepalanchok | 1 |  | 4 | 0 | 0 | 0 |
| 15 | Sindhuli | 0 |  | 4 | 0 | 0 | 0 |
| 16 | Baitadi | 2 |  | 3 | 0 | 0 | 0 |
| 17 | Dhankuta | 1 |  | 3 | 0 | 3 | 0 |
| 18 | Nuwakot | 5 |  | 3 | 0 | 2 | 0 |
| 19 | Rautahat | 7 |  | 3 | 0 | 0 | 0 |
| 20 | Baglung | 2 |  | 2 | 0 | 1 | 0 |
| 21 | Bajhang | 0 |  | 2 | 0 | 0 | 0 |
| 22 | Doti | 3 |  | 2 | 0 | 0 | 0 |
| 23 | Lamjung | 0 |  | 2 | 0 | 1 | 0 |
| 24 | Parsa | 1 |  | 2 | 0 | 0 | 0 |
| 25 | Pyuthan | 0 |  | 2 | 0 | 1 | 0 |
| 26 | Rukum | 0 |  | 2 | 0 | 0 | 0 |
| 27 | Bajura | 0 |  | 1 | 0 | 1 | 0 |
| 28 | Bhojpur | 2 |  | 1 | 1 | 0 | 0 |
| 29 | Gulmi | 0 |  | 1 | 1 | **10** | 0 |
| 30 | Illam | 0 |  | 1 | 0 | 0 | 0 |
| 31 | Jhapa | 0 |  | 1 | 0 | 0 | 0 |
| 32 | Kapilvastu | 0 |  | 1 | 0 | 1 | 0 |
| 33 | Kaski | 0 |  | 1 | 0 | 0 | 0 |
| 34 | Kathmandu | 0 |  | 1 | 0 | 3 | 0 |
| 35 | Mahottari | 0 |  | 1 | 0 | 1 | 0 |
| 36 | Morang | 0 |  | 1 | 0 | 0 | 0 |
| 37 | Palpa | 1 |  | 1 | 0 | **22** | 0 |
| 38 | Ramechhap | 13 |  | 1 | 0 | 1 | 0 |
| 39 | Sunsari | 1 |  | 1 | 0 | 0 | 0 |
| 40 | Syanjga | 0 |  | 1 | 0 | **14** | **2** |
| 41 | Terathum | 0 |  | 1 | 0 | 0 | 0 |
| 42 | Udaypur | 0 |  | 1 | 0 | 0 | 0 |
| 43 | Banke | 0 |  | 0 | 0 | 9 | 0 |
| 44 | Dang | 0 |  | 0 | 0 | 2 | 0 |
| 45 | Salyan | 0 |  | 0 | 0 | 4 | 0 |
| 46 | Argakhachi | 1 |  | 0 | 0 | 2 | 0 |
| 47 | Jajarkot | 0 |  | 0 | 0 | 1 | 0 |
| 48 | Parbat | 0 |  | 0 | 0 | 3 | 0 |
| 49 | Surkhet | 0 |  | 0 | 0 | 2 | 0 |
| 50 | Bardyia | 0 |  | 0 | 0 | 6 | 0 |
| 51 | Khotang | 8 |  | 0 | 0 | 0 | 0 |
| 52 | Darchula | 1 |  | 0 | 0 | 0 | 0 |
| 53 | Siraha | 1 |  | 0 | 0 | 0 | 0 |
|  | **Total** | **141** | **8** | **401** | **14** | **267** | **3** |

**Supplementary Table 2: Characteristic features of animals** captured during scrub typhus outbreak in Nepal

| **Trap ID** | **Rodent (Genus, species)** | **Weight (g)** | **Sex** | **Mammary gland** | **Length (cm)** | | | **Color and pattern** | | | | |
| --- | --- | --- | --- | --- | --- | --- | --- | --- | --- | --- | --- | --- |
|  |  |  |  |  | **Head and body** | **Tail** | **Ear** | **Teeth** | **Hind foot** | **Dorsal fur** | **Ventral fur** | **Tail** |
| RC-5 | *Rattus rattus* | 117.0 | F | 2+3 | 17 | 21 | 2 | Grey | Brown | Dark brown | Brown | Straight and brown |
| RC-7 | *Rattus rattus* | 92.0 | M |  | 17 | 19 | 2 | Grey | Brown | Dark brown | Brown | Straight and brown |
| RC-12 | *Rattus rattus* | 146.6 | M |  | 19 | 12* | 2 | Grey | Brown | Dark brown | Grey | Straight and brown |
| RC-19 | *Rattus rattus* | 84.3 | M |  | 16 | 19 | 2 | Grey | Brown | Dark brown | Grey | Straight and brown |
| RC-33 | *Rattus rattus* | 126.3 | F | 2+3 | 18 | 13* | 2.2 | Grey | Brown | Dark brown | Grey | Straight and brown |
| RC-38 | *Suncus murinus* | 21.2 | F |  | 12 | 5.5 | 0.5 | Sharp and white | Brown | Dark grey | Grey | Straight and brown |
| RC-52 | *Rattus rattus* | 171.0 | M |  | 20 | 18* | 2.2 | Grey | Brown | Dark brown | Grey | Straight and brown |
| RC-64 | *Suncus murinus* | 20.7 | F |  | 11 | 6 | 0.5 | Sharp and white | Brown | Dark grey | Grey | Straight and brown |
| RC-65 | *Rattus rattus* | 128.9 | F | 2+3 | 17.5 | 21 | 2.3 | Grey | Brown | Dark brown | Grey | Straight and brown |
| RC-72 | *Rattus rattus* | 90.0 | M |  | 17 | 19 | 2 | Grey | Brown | Dark brown | Grey | Straight and brown |
| RC-89 | *Rattus rattus* | 125.0 | F | 2+3 | 18 | 16 | 2.1 | Grey | Brown | Dark brown | Grey | Straight and brown |
| RC-97 | *Rattus rattus* | 105.6 | F | 2+3 | 17 | 18.5 | 2 | Grey | Brown | Dark brown | Grey | Straight and brown |

Wt: weight; g: grams; cm: centimeters; M: male; F = female; *part of tail was cut in the trap so that only remaining tail was measured

**Supplementary Table 3: Serological (IFA) and molecular findings of animals and chigger samples** during scrub typhus outbreak in Nepal

| **Trap ID** | **Animal** | **Number of ectoparasitic chigger collected** | **Animal serum IFA** | **Animal tissue PCR** | | **Chigger PCR** |
| --- | --- | --- | --- | --- | --- | --- |
|  |  |  |  | **Lung** | **Liver** |  |
| RC-5 | *R. rattus* | ND | Negative | Negative | Negative | Negative |
| RC-7 | *R. rattus* | **4** | **Positive** | Negative | Negative | **Positive** |
| RC-12 | *R. rattus* | ND | Negative | Negative | Negative | Negative |
| RC-19 | *R. rattus* | ND | Negative | Negative | Negative | Negative |
| RC-33 | *R. rattus* | **3** | **Positive** | Negative | Negative | Negative |
| RC-38 | ***S. murinus*** | ND | Negative | Negative | Negative | Negative |
| RC-52 | *R. rattus* | ND | Negative | Negative | Negative | Negative |
| RC-64 | ***S. murinus*** | ND | Negative | Negative | Negative | Negative |
| RC-65 | *R. rattus* | ND | Negative | Negative | Negative | Negative |
| RC-72 | *R. rattus* | **3** | **Positive** | Negative | Negative | Negative |
| RC-89 | *R. rattus* | ND | Negative | Negative | Negative | Negative |
| RC-97 | *R. rattus* | ND | Negative | Negative | Negative | Negative |

IFA, indirect fluorescence antibody assay; PCR, polymerase chain reaction; ND, not detected;
